# Publication practices of medical students at medical schools in Palestine: experiences, attitudes, and barriers to publishing

**DOI:** 10.1097/MS9.0000000000002372

**Published:** 2024-07-15

**Authors:** Afnan W.M. Jobran, Jehad Feras AlSamhori, Moath Rushdi Atyani, Mohammed Abdulrazzak, Zarmina Islam, Sifwa Safdar, Shoaib Ahmad, Hassam Ali

**Affiliations:** a Faculty of Medicine, Al Quds University, Jerusalem; b Faculty of Medicine, Hebron University, Hebron, Palestine; c Faculty of Medicine, University of Jordan, Amman, Jordan‬; d Faculty of Medicine, University of Aleppo, Aleppo, Syria; e Faculty of Medicine, Dow Medical College, Dow University of Health Sciences, Karachi, Sindh; f Allama Iqbal Medical College, Lahore; g Punjab Medical College, Faisalabad, Pakistan; h Department of Internal Medicine, ECU Health Medical Center/Brody School of Medicine, Greenville, NC, USA

**Keywords:** barriers to publishing, medical schools, medical students, Palestine, publication practices

## Abstract

**Introduction and importance:**

The progression in research and studies made by each nation’s scientific communities serves as one of the primary metrics for evaluating that nation’s scientific development; in this study of medical students at Palestinian medical colleges, attention was given to individuals who had been involved in research activities during their undergraduate training.

**Materials and methods:**

The authors conducted an online survey among medical students in Palestinian medical institutions, focusing on those engaged in research processes throughout their undergraduate studies. The study started in March 2022 and ended in the final week of May 2022. Participants were instructed to respond to statements regarding research work in the online self-administered questionnaire.

**Results:**

In the authors’ study, out of 425 participants, only 4.9% published an article. There were no significant gender disparities between males and females who published at least one article. There was a strong association between the year of study and publishing, with clinical students being more likely to publish (90.4%) than preclinical students (8.6%). The authors’ study revealed that students published either to enhance their curriculum vitae (33.0%) or out of personal interest (19.0%).

**Conclusion:**

While participants in the authors’ study demonstrate high levels of awareness and favorable attitudes toward research, active participation in the research community is still insufficient. More opportunity and mentorship are among the stated obstacles to participation in research. To overcome these obstacles, the authors suggest making long-term investments in research training, starting research clubs, and offering coaching and mentorship.

## Introduction

Highlights

- A cross-sectional study was conducted among medical students in Palestinian medical institutions, focusing on undergraduate studies.
- In our study, out of 425 participants, only 4.9% published an article, which was lower than other studies.
- Our study found that the most common reasons for a lower rate of publication by medical students were a lack of opportunity, guidance and supervision, and insufficient time.

The progression in research and studies made by each nation’s scientific communities serves as one of the primary metrics for evaluating that nation’s scientific development^1^. The amount and quality of research articles are essential determinants of the advancement of science in any nation^2^. It is crucial to recognize and remove the obstacles that stand in the way of scientific research’s advancement because it is a crucial part of a nation’s growth, development, and status.

Research is a systematic process to acquire new knowledge, science, or invention through standardized methods. It involves the creation of new knowledge or the application of existing knowledge in novel ways to generate fresh concepts, methodologies, and understandings and achieve new results. Research in medicine and clinical practice is critical to improving healthcare services. All aspects of healthcare and medicine, including disease surveillance, treatment, diagnosis, and prevention, heavily rely on the quality of medical research. Since universities are most countries’ primary sources of scientific research, medical students play a significant role in research productivity^3^.

It has been shown that participating in research experiences helps medical students improve their ability to communicate data and findings, understand literature, and think critically. According to studies, students who conduct research early in their schooling are more inclined to do so again later in their careers^3,4^. These benefits can explain the growing competition among doctors for jobs, as research has become an essential form of assessment for career and personal development.

Earlier studies have recognized several elements as impediments to research in diverse societies. The main obstacles to research by medical students are time limits in medical schools, a lack of financing support, and a need for an understanding of study design or result interpretation^1^. Lack of research opportunities and training, lack of confidence in one’s ability to carry out a successful study (lack of research self-efficacy), inadequate support from professors, waning interest in research, and limited access to data sources (such as the internet), materials, and equipment are other issues that have been cited as barriers^1^. According to studies conducted at one University, introducing a formal research workshop dramatically increased the number of student submissions of publications (from 11 to 59%)^5^.

A study assessing the quantity and quality of medical and biomedical research published in Palestine between 2002 and 2011 identified 770 publications in the medical and biomedical fields, averaging ~80 articles per year^6^. Another study, a systematic review of the quality of reports on medical and public health research in Palestinian institutions, concluded that the quality of Palestinian medical and health reports has improved but still needs to improve at a satisfactory level. The authors attribute this subpar reporting to increased waste in research and a reduction in research value^7^.

This study aims to examine the experiences, motivations, attitudes, and particularly the barriers to research among medical students at medical schools in Palestine, focusing on promoting research as per The STROCSS 2021 Guideline^8^.

## Materials and methods

The survey was conducted among medical students in Palestinian medical institutions, focusing on those engaged in research processes throughout their undergraduate studies. The objective was to learn more about Palestinian medical students’ research paper writing and publication practices, the challenges they had in having their work published, and the elements that either helped or impeded the research process in Palestine. Several medical schools in Palestine participated in the survey, including those in the West Bank and Gaza. The study, which started in March 2022 and ended in the final week of May 2022, involved MBBS (Bachelor of Medicine and Bachelor of Surgery), and MD (Doctor of Medicine) students from these universities.

The study included all MBBS and MD students from every region within Palestinian medical institutions. This criterion aimed to encompass a wide range of students from different geographical areas to enhance the representativeness of the sample, which was anticipated to be around 1500 students—making 50% of the MBBS and MD students admitted each year into Palestine’s public sector. Graduated students, resident doctors, and other healthcare providers were excluded from the study. This exclusion criterion was important to focus specifically on undergraduate medical students engaged in research processes during their studies.

The Institutional Review Board approved the study and waived the need for written informed consent because completing the survey questionnaire constituted implied consent. All personal information was kept private, participation was voluntary, and anonymity was guaranteed. Researchers analyzed only de-identified data to ensure anonymity.

Participants were required to respond to a questionnaire distributed to them in one of two ways: Online Google Forms and Print media. The first technique asked participants to select the most suitable response to each topic using an online application for data collecting. Afterward, participants were contacted via official and informal networks to participate in the study. The communication also contained an invitation outlining the goals of the study, the name and contact details of the principal investigator, and a direct link to the survey platform (Google Forms).

Participants were instructed to respond to statements regarding research work, publication procedures, and associated challenges in the online self-administered questionnaire. Additionally, a site coordinator at each medical institution provided weekly verbal reminders via phone calls and face-to-face interactions. Only the complete responses were recruitment to the statistical analysis.

The survey was developed based on existing literature and then customized to suit the specific needs and characteristics of the Palestinian medical student population. To ensure the validity and reliability of the survey instrument, rigorous steps were taken to verify its appropriateness for use in the local context. This validation process involved consulting with multiple experts in the field of medical education and research in Palestine to review the survey items, assess their relevance, and provide feedback on the clarity and appropriateness of the questions. By involving local experts in the validation process, the study aimed to enhance the quality and relevance of the survey instrument and mitigate potential biases associated with self-reporting and response rates.

Upon completion of the study period, the online data were downloaded from the survey tool, entered into an Excel spreadsheet, and anonymized. Statistical software was then utilized to import the data for analysis. Descriptive analyses were performed to examine the baseline characteristics of participants and outcome variables. Means with standard deviations (SD) were reported for continuous variables using *t*-test, while frequencies and percentages were generated for categorical variables using Pearson’s χ^2^ test (N%). The second method involved using a printed version of the Google Form. Participants filled it out according to their preferences and submitted it to the survey administrators. This information was manually entered into the online Google Forms, downloaded, input into Excel spreadsheets, and then analyzed in a manner like the first method.

A well-designed and authorized questionnaire was employed for data collection. Data analysis was conducted using the Statistical Package for Social Sciences (SPSS) version 24. The data were collected using a questionnaire with closed-ended or semi-structured response options.

### Ethical considerations

Participants’ permission was requested before any data were gathered. The study’s aims were explained, and confidentiality was guaranteed. The ethical review committee authorized the questionnaire, and prior approval was acquired to conduct the study. The study’s goals were explained to the participating medical students from various universities.

## Results

The average age of the participants was 20.36±1.6 years. Females were more prevalent than males (53.2% vs. 46.8%). The largest proportion of respondents were second-year students (34.4%) and third-year students (26.6%). Fourth- and fifth-year students comprised 28% of the participants. Out of 425 participants, only 4.9% had published an article. Among the 21 participants involved in publishing, 20% had published one article, while the remaining had published more than one article (Table 1).

**Table 1:** Summary of the socio-demographic characteristics of 425 study participants

Variables	No. students, *n* (%)
Age (mean±SD)	20.36±1.6
Sex	
Male	198 (46.8)
Female	227 (53.2)
Year of education	
1st year	42 (9.9)
2nd year	146 (34.4)
3rd year	113 (26.6)
4th year	87 (20.5)
Final year	32 (7.5)
Graduate	5 (1.2)
Articles published	
Yes	21 (4.9)
No	404 (95.1)

There were no statistically significant differences across ages and genders in terms of publishing an article (*P*>0.05). Medical students in their fourth year or higher published articles more frequently than preclinical medical students (first through third year) (*P*<0.001) (Table 2).

**Table 2:** Characteristics of 21 study participants who published in a journal

Variables	No. students, *n* (%)	*P*
Age (mean±SD)	21.0±1.67	0.372
Sex		
Male	9 (42.9)	0.227
Female	12 (57.1)	
Year of education		
1st year	0	<0.001
2nd year	1 (4.8)	
3rd year	1 (4.8)	
4th year	7 (33.3)	
Final year	7 (33.3)	
Graduate	5 (23.8)	

**Figure 1:**
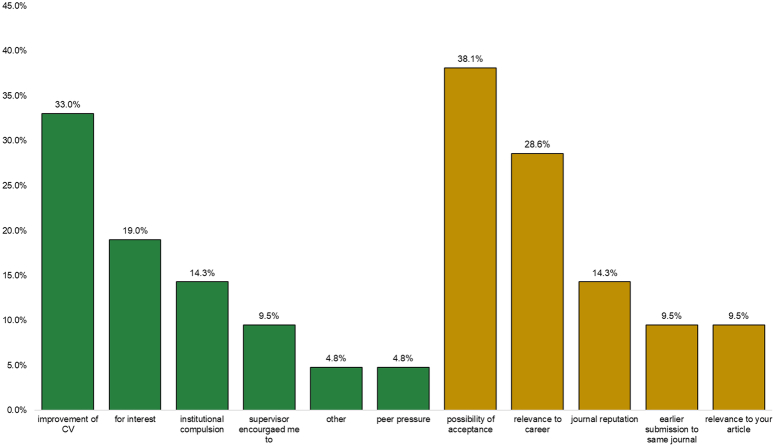
Reasons for publication and choice of journal.

The primary reason for publishing was to enhance their curriculum vitae (CV) (33.0%), followed by a personal interest in research (19.0%). Encouragement from supervisors constituted another major reason for publishing (14.3%), while peer pressure and other reasons accounted for the rest (6.6%). The main driving factor behind choosing a particular journal was the possibility of acceptance (38.1%), followed by the relevance to their career and the reputation of the journal (26.6% and 14.3% respectively) (Fig. 1).

Letters, abstracts, and other article types comprised about 20% of the total published publications (20.3%, 19.4%, and 19.4%, respectively). Co-authors made up the majority of paper authors among participants (32.4%), followed by first and second authors (17.6% each) and third authors (22.2%) (Table 3). Only 20.4% of articles were accepted without revision, whereas approximately 29.6% were accepted after revision. About 22.2% of the articles were desk rejected, and 27.8% required revision at the time of the survey (Table 4).

**Table 3:** Type of articles published and the rank of authors

	First author, *n* (%)	Second author, *n* (%)	Third author, *n* (%)	Fourth author, *n* (%)	Other authors, *n* (%)	Total
Original paper	7 (33.3)	5 (23.6)	1 (4.8)	4 (19.0)	0	17 (15.7)
Review	1 (4.8)	5 (23.8)	4 (19.0)	1 (4.8)	2 (9.5)	13 (12.0)
Case report	2 (9.5)	5 (23.8)	4 (19.0)	2 (9.5)	1 (4.8)	14 (13.0)
Letter	1 (4.8)	2 (9.5)	6 (28.9)	2 (9.5)	11 (52.4)	22 (20.3)
Abstract	3 (14.3)	5 (23.8)	3 (14.3)	1 (4.8)	9 (42.9)	21 (19.4)
Other	5 (23.8)	2 (9.6)	1 (4.8)	1 (4.8)	12 (57.1)	21 (19.4)
Total	19 (17.6)	24 (22.2)	19 (17.6)	11 (10.1)	35 (32.4)	108

**Table 4:** Publication acceptance rates by category

	Accepted with revision, *n* (%)	Accepted without revision, *n* (%)	Rejected outright, *n* (%)	Revision in progress, *n* (%)	Total
Original paper	8 (25.0)	5 (22.7)	2 (8.3)	2 (6.7)	17 (15.7)
Review	3 (9.4)	4 (18.1)	3 (12.5)	3 (10.0)	13 (12.0)
Case report	7 (21.9)	3 (13.6)	1 (4.2)	3 (10.0)	14 (13.0)
Letter	6 (18.8)	5 (22.7)	2 (8.3)	9 (30.0)	22 (20.3)
Abstract	3 (9.4)	3 (13.6)	8 (33.3)	7 (23.3)	21 (19.4)
Other	5 (15.6)	2 (9.0)	8 (33.3)	6 (20.0)	21 (19.4)
Total	32 (29.6)	22 (20.4)	24 (22.2)	30 (27.8)	108

**Figure 2:**
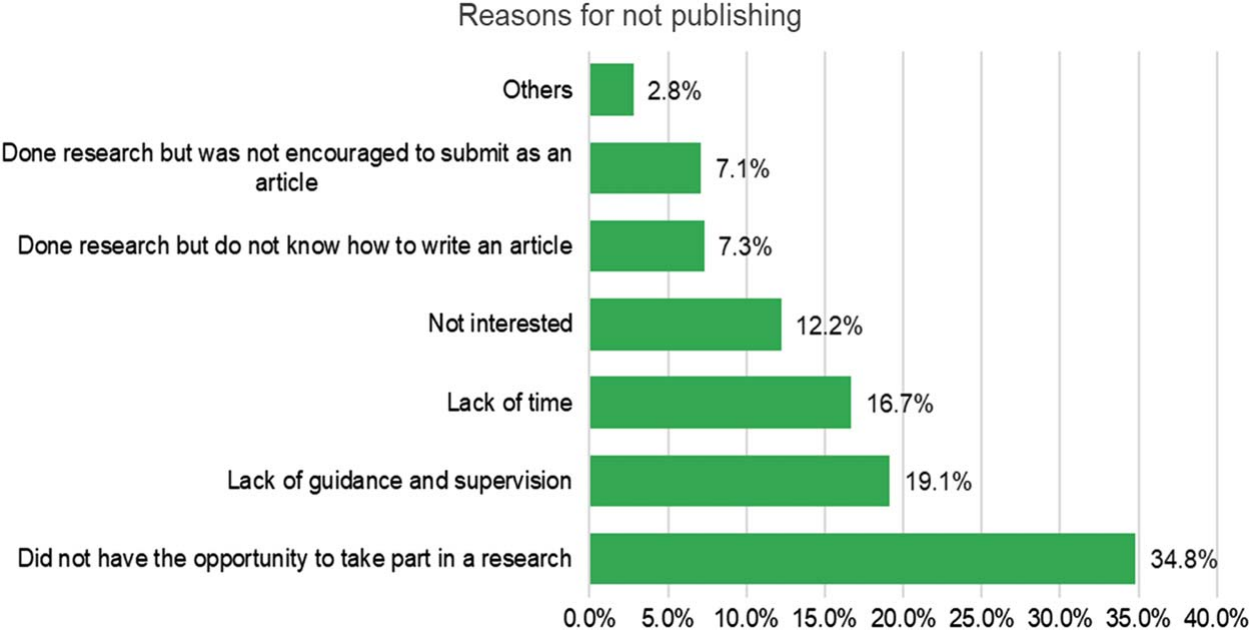
Reason for not publishing among the study population.

The majority of participants (95.1%) had never published an article before. The main reasons for this included lack of opportunity (34.8%), lack of guidance (19.1%), insufficient supervision and time (16.7%), and lack of interest in publishing (12.2%). Additionally, 14.3% of respondents had previously conducted research but either needed to learn how to write an article (7.3%) or were not given enough encouragement (7.3%) (Fig. 2).

A total of 86.8% of participants expressed a desire to engage in research, and 76.2% believed that publishing involvement was crucial. Gaining experience and strengthening their resume were cited as the top reasons for considering publishing papers (77.8% and 23.2%, respectively). In terms of specific research interests, 3.8% were solely interested in audits, 5.9% were solely interested in research, and the majority were interested in both audits and research projects.

The majority of respondents (64.1%) indicated a preference for receiving more instruction on writing abstracts and different types of papers. More than half reported not receiving instruction on writing an abstract or a paper (59.1% and 50.8%, respectively), and 65.2% stated that they were unable to critically evaluate a work. Concerning the steps involved in the publication, 60.1% indicated a lack of knowledge, while more than half expressed an interest in learning more (61.6%).

## Discussion

In our study, out of 425 participants, only 4.9% published an article, which was lower than other studies, such as 14% in Britain^9^, 15% in Sweden^10^, and 22.5% in Uganda^11^. There were no significant gender disparities between males and females who published at least one article in our study. This finding aligns with existing data and various proposed explanations for the “gender gap” discussed by Guelich, J.M., who concluded that women now constitute a larger percentage of first and senior physician authors of original research in the United States than they did forty years ago^12^.

Understanding the research culture, available resources, and institutional support for research within medical schools in Palestine is crucial for a comprehensive analysis of medical education and research practices in the region. The absence of detailed information on these aspects in research studies can limit the depth of interpretation of findings and potentially overlook key factors influencing students’ attitudes and experiences with research. By shedding light on the research environment in Palestinian medical schools, including the integration of research into the curriculum, the availability of research opportunities, faculty involvement, and institutional support mechanisms, researchers can provide a more nuanced understanding of the context in which medical students engage in research activities. This contextual information is essential for contextualizing study results and recognizing the challenges and opportunities that students face in their research endeavors during their medical training.

There was a strong association between the year of study and publishing, with clinical students being more likely to publish (90.4%) than preclinical students (8.6%). This trend has been observed in previous literature; for example, a study from Britain showed that 70% of the respondents who had published before were in their clinical years^9^. Similarly, in Uganda, it was found that compared to first-year students, participants in clinical years had higher odds of engaging in research^11^. This result is consistent with findings by Kyaw and collaborators from Malaysia, where preclinical students were less aware of research than clinical students^13^.

This trend may be attributed to students in more advanced study levels having greater experience with different health sectors, including research course units, and increased opportunities to observe and interact with senior researchers in the field. This exposure raises the likelihood of students finding inspiration, mentorship, and collaboration for ongoing research.

Furthermore, students in their later academic years have grown accustomed to the demands of medical school, making it easier for them to balance academics with extracurricular activities, including research^11^. As for the motivation to publish, our study revealed that students published either to enhance their CV (33.0%) or out of personal interest (19.0%). Students may have various other motivations and external influences that drive their publishing decisions. These could include factors such as academic pressure, peer influence, faculty encouragement, career advancement opportunities, financial incentives, or institutional requirements. Further research could delve deeper into these additional motivations to provide a comprehensive understanding of the complex factors influencing students’ decisions to publish. In line with this, Bonilla-Escobar emphasizes the importance of providing research training in a comprehensive and multidisciplinary manner, considering that students’ interest in a particular field plays a crucial role in sparking their curiosity in research. Having passionate teachers was one of the most influential factors in research motivation. This approach ensures that all medical and health workers develop the capacity for critical thinking and analysis^14^.

Additionally, Pathipati A.S. and his team discovered that while many students (23%) claimed “academic interest” as their primary motivation for taking time off for research, these students were a significant minority^15^. Most students base their decisions on other factors. Moreover, students’ motivation varied depending on the competitiveness of their chosen specialty. The most common reason given by students for taking a research year was to “increase competitiveness for residency application” (32%). Numerous residency programs consider research a critical component in their selection process, and this emphasis has only grown over time^15^.

In terms of factors influencing the choice of journal, our study found that the likelihood of acceptance (38.1%), career relevance (26.7%), and journal reputation (14.3%) were the most significant factors. A study in Uganda revealed that 70% of individuals who submitted articles did so in international publications rather than local or regional ones^11^. This preference may stem from the fact that more international journals than regional ones waive article processing charges (APCs), either in whole or in part, for authors from low-income countries. Another probable reason is the perceived higher recognition and appreciation of authors in international journals than local and regional ones^11^. Furthermore, authors in resource-constrained settings have previously reported APCs as one of the primary barriers^16^.

To reduce bias based on the author’s educational background, high-impact journals should allocate sections exclusively for student submissions, as students often worry about rejection due to journals favoring experts in the field^17^. Concerning authorship, most of our participants who had published were second authors (22.2%), followed by first (17.6%) or third authors (17.6%). In the Uganda study, 39 (25.3%) published as first authors, and 25 (16.2%) as co-authors^11^. Additionally, a Saudi study revealed that 47.4% presented their research, with 11.8% as co-authors^18^. Approximately 28% of papers published in Medline-indexed journals have students listed as co-authors, according to academic supervisors at a German institution^19^.

A study suggests that students can be highly involved in research projects when provided with the right conditions and motivation. Senior physicians should consider involving enthusiastic students in their extracurricular or elective research. Additionally, medical educators should recognize the value of student research and, if possible, integrate opportunities into the curriculum^20^.

Regarding reasons for not publishing, our study found that the most common obstacles were a lack of opportunity (34.8%), guidance and supervision (19.1%), and insufficient time (16.7%). A significant hurdle for individuals who have yet to publish is the lack of research opportunities. Lack of research opportunities was found to be a significant hurdle for individuals who have yet to publish. This could be due to limited funding or resources, a lack of available research projects, or a competitive environment where only a select few students have access to research opportunities. In addition, the lack of supervision and insufficient time were also reported in studies conducted in Pakistan, which indicates that student research has difficulties. For instance, good mentoring can lead to satisfaction with research. Other issues include the need for more time, the neglect of routine investigations, the decline in clinical abilities is due to more time spent on research activities and the need for adequate project management^21,22^. Moreover, a Saudi study discovered that the second significant barrier preventing male medical students from conducting research is a lack of support from mentors or assistants. The impact of these hurdles is apparent in 95.1% of our participants had never participated in a research study or an audit^23^.

To overcome these barriers and promote research participation among students, it is crucial to implement potential solutions. One possible solution is to establish mentorship programs where experienced researchers can guide and support students in their research endeavors. These mentorship programs can provide valuable guidance on research methodologies, literature reviews, data analysis, and manuscript writing. Additionally, universities and institutions can allocate more resources and funding toward research initiatives, creating more opportunities for students to engage in research activities.

Another significant finding in our study was the substantial gap in research paper writing instruction received by students. Only 34.8% of our participants knew how to write a paper critique, which is lower than the 49% reported by Griffin, who believed they were skilled paper critics. The medical curriculum should incorporate lessons that teach students this essential skill. This equips medical students with the tools to differentiate between reliable and false information in an era where information is readily accessible, enabling them to make informed decisions about patient care^9^.

Additionally, despite embracing such instruction, 50.8% and 59.1% of respondents stated they had not received training on writing papers or abstracts, respectively, which is also lower than Griffin’s findings^9^, where 70% and 78% acknowledged they had not been taught how to write abstracts and articles, respectively. Only a few studies, like the one by Jackson^24^, have demonstrated that providing writing instruction can increase publication output. Jackson highlights that the extensive and ongoing mentoring and support from experienced writers helped novice writers. Benefits to the organization included increased team and research higher degree student participation in publication activities, improved collegial connections, and opportunities for senior established writers to collaborate with novice writers.

Medical students who completed courses in research methodology are more motivated to address scientific issues and pursue academic careers^25^. According to our research, the majority (64.1%) expressed interest in receiving additional instruction on creating abstracts and different types of articles. Regarding the publication process, 60.1% were unaware of the steps, and over half (61.6%) wanted to learn more. This finding aligns with Griffin’s study, where 86% of medical students believed it was essential to publish research, and 90% thought they would benefit from instruction on writing papers, 87% on creating abstracts, and 91% on publishing practices^9^.

Furthermore, our study indicates that 86.8% of participants would like to engage in research, and 76.2% believe it is important to be involved in publishing research. These numbers are similar to those reported by Griffin, who stated that 86% of respondents would appreciate more opportunities to participate in research or audits^9^. Only 52% of students expressed interest in participating in more laboratory research, compared to 91% who preferred more clinical research. Gaining experience (77.8%) and improving their CV (23.2%) were the primary motivations for considering publishing papers. Additionally, when considering both students who had submitted articles and those who had not, students felt it was essential to publish to advance their careers^9^.

To enhance medical students’ perceptions of research, we suggest providing motivating materials and mentorship, encouraging medical students to engage in the publishing process by creating a supportive environment and availability of research-related courses to help students acquire additional knowledge and research-related skills. Moreover, initiating research journal clubs could offer medical students and their teaching doctors’ opportunities to engage in productive discussions. Lastly, colleges could collaborate with international journals to expedite publishing by reducing publication costs.

This study has several strengths, including the large population of Palestinian medical students, participation from multiple universities, examination of medical students across all academic years, and efforts to analyze potential barriers to publishing practices. Additionally, we developed a survey that can be successfully implemented in medical schools in the future to evaluate medical students’ publication activities. However, there are certain limitations, as we used a convenience sampling method, and only responses from respondents who completed the online survey were collected. They may only be representative of some continuing medical students in the country. Moreover, the results rely on self-reported answers from participants without formal verification by the researchers, such as the total number of papers published and the journals read, making them subject to recall bias and inaccuracies. Nevertheless, as a nationwide study encompassing all medical schools and their programs in the country, with significant participation from each school, these findings can be generalized.

## Conclusion

Our study with 425 participants showed only 4.9% had published an article, much lower than in other countries. No significant gender disparities were found in publication rates. Clinical students were significantly more likely to publish than preclinical ones, consistent with international trends. Motivations for publishing included enhancing CVs and personal interest, while lack of opportunities and guidance were major obstacles. The study suggests that mentorship, increased funding, and research training are crucial for improving research participation. Limitations include the convenience sampling method and reliance on self-reported data, but the findings are broadly generalizable across Palestinian medical schools. By fostering a culture of research engagement and providing support for students to actively participate in research activities, institutions can unlock the full potential of their student community and contribute to advancements in healthcare and scientific development in the region.

## Ethical approval

The study was approved by the Ethics committee of Faculty of Medicine in our university. A copy of the Ethical Approval is available for review by the Editor-in-Chief of this journal on request.

## Consent

Participants’ permission was requested before any data were gathered. The study’s aims were explained, and confidentiality was guaranteed. Written informed consent was obtained from the participants for publication. A copy of the written consent is available for review by the Editor-in-Chief of this journal on request.

## Source of funding

Not applicable.

## Author contribution

All authors fulfill the authorship criteria because of their substantial contributions to the conception, design, analysis, and interpretation of the data. A.W.M.J., J.F.A. and M.R.A. administrated the project, researched literature, wrote the first draft of the manuscript. A.W.M.J. and S.A. conceptualized the study, prepared the survey materials, questionnaire design, coordinate and monitor the data collection process with collaborators, and assisted in ethical approval proposal. M.A. reviewed the study, manuscript editing and participated in writing the final version. Z.I. and S.S. participated in organizing and data arrangement, coded data, involved in statistical analysis, and also participated in the final table’s design and assisted in ethical approval proposal. H.A. supervised all steps, checked writing, and approved methodology. All authors read and approved the final manuscript.

## Conflicts of interest disclosure

The authors declare that they have no competing interests.

## Research registration unique identifying number (UIN)

Research Registry researchregistry10148.

https://researchregistry.knack.com/researchregistry#home/registrationdetails/66075ecfcc9bc10027ec3bc2/.

## Guarantor

Afnan W.M. Jobran.

## Data availability statement

The datasets generated and analyzed during the current study are not publicly available.

## Provenance and peer review

Not commissioned, externally peer-reviewed.
